# Sequential Treatment with Pazopanib and Everolimus in Metastatic Renal Cell Carcinoma

**DOI:** 10.3389/fphar.2017.00484

**Published:** 2017-07-20

**Authors:** Sabrina Rossetti, Carmine D'Aniello, Gelsomina Iovane, Sarah Scagliarini, Maria M. Laterza, Fernando De Vita, Clementina Savastano, Giacomo Cartenì, Maria A. Porricelli, Massimiliano Berretta, Salvatore Pisconti, Gaetano Facchini, Carla Cavaliere

**Affiliations:** ^1^Division of Medical Oncology, Department of Uro-Gynaecological Oncology, Istituto Nazionale Tumori Fondazione G. Pascale (IRCCS) Naples, Italy; ^2^Division of Medical Oncology, Ospedali dei Colli-Monaldi-Contugno-CTO, Azienda Ospedaliera Specialistica Dei Colli (AORN) Naples, Italy; ^3^Unità Operativa Sperimentazioni Cliniche Oncologia, Azienda Ospedaliera di Rilievo Nazionale ‘Antonio Cardarelli,’ Naples, Italy; ^4^Division of Medical Oncology, Department of Internal and Experimental Medicine, “F. Magrassi” Second University of Naples Naples, Italy; ^5^Unità Operativa Oncologia, Azienda Ospedaliera Universitaria OO.RR. San Giovanni di Dio Ruggi d'Aragona Salerno, Italy; ^6^Department of Medical Oncology, Centro di Riferimento Oncologico (CRO), National Cancer Institute Aviano, Italy; ^7^Department of Onco-Hematology Medical Oncology, S.G. Moscati Hospital of Taranto Taranto, Italy; ^8^Operating Unit Complex (U.O.C.) of Medical Oncology Nola, Italy

**Keywords:** metastatic renal carcinoma, pazopanib, everolimus, sequential therapy, real-world

## Abstract

In metastatic renal cell carcinoma, complete response to first-line antiangiogenic agents is rare and resistance to therapy often develops. Protocols for sequential treatment with angiogenesis and mTOR inhibitors are under evaluation to improve outcomes. In this observational, real-world study, patients received a first-line therapy with pazopanib until discontinuation for disease progression or toxicity, then a second-line with everolimus. Primary endpoints were overall survival (OS) for sequence, progression free survival (PFS) for each agent, and safety. Thirty-one patients were included in the analysis: 73.3% of patients underwent nephrectomy before treatment, 25.8% had at least three comorbidities. At the beginning of therapy, the median age was 68 years, with more than 60% of patients older than 65 years. The median OS for sequence was 26.5 months (95% CI 17.4-nc); median PFS was 10.6 months (95% CI 6.3–12.1) with pazopanib and 5.3 months (95% CI 3.8–6.7) with everolimus. The median persistence in pazopanib therapy was 8.1 months (Interquartile Range IQR 5.3–12.7), with 31% of patients who required dose reduction, while persistence in everolimus was 4.4 months (IQR 3.4–6.5). Sequence was well tolerated with a different profile of adverse events for each agent. These data confirmed that pazopanib was effective, even in reduced dosing, and well tolerated and suggested that everolimus may represent an opportunity to continue a therapy when patients cannot further tolerate angiogenesis inhibitors or develop a resistance.

## Introduction

Sequential treatment in metastatic renal cell carcinoma (mRCC) is of interest as a complete response to treatment with first-line antiangiogenic agents is rare and tyrosine kinase inhibitors usually do not produce long term remission: patients relapse when therapy is discontinued, or a resistance develops during treatment (Facchini et al., 2009; Albiges et al., 2012; Duran et al., 2017). Sequential therapies with targeted agents should be considered in all patients who can tolerate these treatments: level 1 evidence supports sequential use of VEGFR inhibitors, followed by everolimus (Escudier et al., 2012). Efficacy and safety of everolimus (10 mg/day) after progression on sunitinib and/or sorafenib compared to placebo was firstly established in the RECORD-1 (Renal Cell cancer treatment with oral RAD001 given daily) randomized trial (Motzer et al., 2010). The median progression free survival (PFS) was 5.5 months (by investigators) compared to 1.9 month with placebo (HR 0.32, *p* < 0.001), while the median overall survival (OS) was 14.8 months (everolimus) vs. 14.4 months (placebo) (HR = 0.87; *p* = 0.162), with 80% of patients in the placebo arm crossed-over to everolimus (Motzer et al., 2010). In real world setting, stable disease in 62% of patients and partial response in 19% of patients were achieved in a second-line treatment with everolimus after failure of tyrosine kinase inhibitors (Rizzo et al., 2015). A recent retrospective review of medical charts of patients with mRCC in the US indicated that sunitinib and everolimus were the most commonly-used first and second targeted therapies, respectively; the use of pazopanib as first targeted therapy, and of axitinib and sorafenib as second targeted therapies, increased over time (Pal et al., 2017a). However, no significant differences among outcomes while receiving second targeted therapy with everolimus for patients treated with pazopanib vs. sunitinib/sorafenib as first targeted therapy resulted from another retrospective analysis of medical charts (Pal et al., 2016). A median OS of 16 months and a median PFS of 5.7 months were achieved by everolimus or temsirolimus after progression with first-line pazopanib (Vogelzang et al., 2015). An alternating treatment with pazopanib and everolimus vs. continuous pazopanib was explored to delay the disease progression in naïve patients with mRCC; no significant differences in prolonged PFS, fewer toxic effects, or improved quality of life, were observed in the alternating treatment and the first-line treatment with a VEGF inhibitor remained the optimal approach in mRCC (Cirkel et al., 2016).

This retrospective, observational study would describe the clinical outcomes of the pazopanib and everolimus sequential therapy in unselected patients with mRCC.

## Materials and methods

### Study design

This multi-centric, real-world, observational study included consecutive patients who were newly diagnosed with mRCC and received a sequential therapy with pazopanib followed by everolimus. Pazopanib treatment was continued until disease progression or discontinuation for toxicity; then, everolimus was given until discontinuation for toxicity, progression, or death. The study was approved by the Istituto Nazionale Tumori - IRCCS “Fondazione G. Pascale” ethical committees and a written informed consent was signed by all subjects, according to the Declaration of Helsinki (no trial registration number available).

### Statistical analysis

The primary endpoints were OS for sequence and objective response rate (ORR), disease control rate (DCR), and PFS for each treatment. The safety profile for each agent was also evaluated for all patients who received at least one dose of drugs. The potential relationships between baseline characteristics and response were even explored. PFS was defined as the interval between the date of the first dose of drug (either pazopanib or everolimus) and the date of disease progression or death for any cause (Cecere et al., 2016); disease progression was defined as radiological tumor progression according to Response Evaluation Criteria In Solid Tumors, RECIST (Vogelzang et al., 2015) version 1.1, or clinical progression or death. AEs were graded according to Common Terminology Criteria for Adverse Events version 4.0.

Data were shown as mean and standard deviation or as mean and 95% CI or as absolute (n) and relative frequency (%). Baseline characteristics and variables distribution were compared using a Chi-square test for categorical variables and a Student *t*-test for continuous variables. All *p*-values are 2-sided and the minimum level of statistical significance was set at *p* < 0.05. Univariate Kaplan-Meier survival analysis was used to estimate survival and generate survival curves and was applied to all time-to event variables (PFS and OS). A time-dependent Cox proportional-hazard regression model was used to compare time-to-event variables; hazard ratios (HRs) and 95% confidence intervals (95% CIs) were calculated. All statistical analyses were performed using JMP statistical software version 13.0 (SAS Institute, Cary, NC).

## Results

### Baseline characteristics

**Figure 1:**
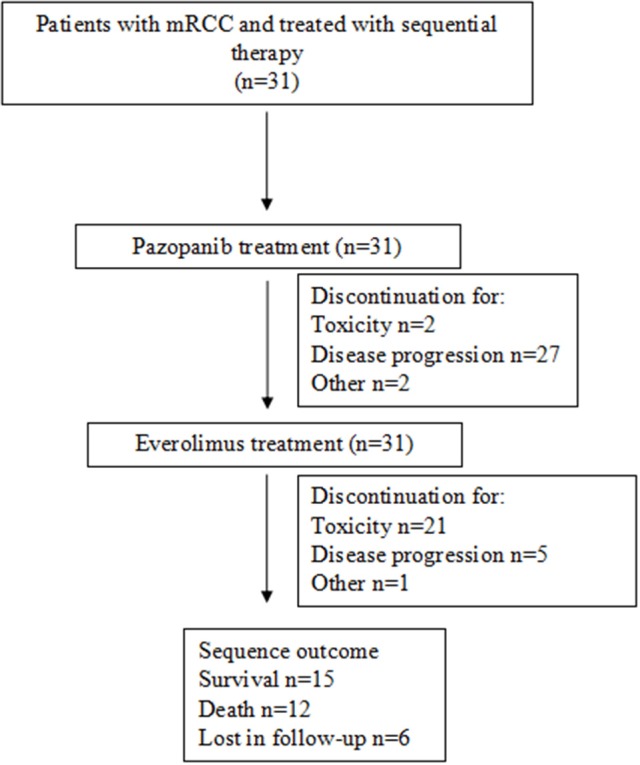
Study overview.

From July 2012 to April 2016, 31 patients with mRCC started a sequential therapy with pazopanib and everolimus and were included in the analysis (Figure 1). Twenty-one patients (67.7%) were men and 22 (73.3%) underwent nephrectomy before starting pazopanib. At the beginning of sequence, the median age was 68 years, with more than 60% of patients older than 65 years. Four patients did not have any comorbidities, 19 (61.3%) had >3 comorbidities, 7 (22.6%) had 3–4 comorbidities and one patient had more than 4 comorbidities; hypertension was the most frequently observed comorbidity (*n* = 12, 38.7%). Clear cell histology was diagnosed in 27 patients, including 2 patients with clear cell and sarcomatoid cell; two patients had a type I papillary tumor; two had a type II papillary tumor. Other baseline characteristics were considered at the beginning of pazopanib and everolimus treatment and are summarized in Table 1. The proportion of patients with both Karnofsky Performance Status (KPS)<80 and Eastern Cooperative Oncology Group (ECOG)>1 was significantly higher during everolimus treatment than during pazopanib treatment; age, risk status [both MSKCC (Motzer et al., 2002) and IMDC (Heng et al., 2009, 2013)] and metastasis sites did not significantly change during the sequence (Table 1).

**Table 1:** Baseline characteristics.

**Characteristics**	**Pazopanib (*n* = 31)**	**Everolimus (*n* = 31)**	***P*-value**
Age	68.0 (8.0)	68.8 (7.9)	*P* = 0.68
**AGE GROUP**			
			*P* = 0.59
<65	12 (38.7)	10 (32.3)	
≥65	19 (61.3)	21 (67.7)	
**ECOG**			
0	17 (54.8)	6 (19.4)	*P* = 0.007
1	10 (32.3)	22 (71.0)	
2	4 (12.9)	2 (6.4)	
3	–	1 (3.2)	
**KPS**			
<80	7 (22.6)	17 (54.8)	*P* = 0.002
≥80	24 (77.4)	11 (35.5)	
Unknown	–	3 (9.8)	
**RISK STATUS MSKCC**			
Good	6 (19.4)	2 (6.5)	*P* = 0.30
Intermediate	21 (67.7)	22 (71.0)	
Poor	4 (12.9)	6 (19.4)	
Unknown	–	1 (3.2)	
**RISK STATUS IMDC**			
Good	6 (19.4)	2 (6.4)	*P* = 0.22
Intermediate	21 (67.7)	22 (71.0)	
Poor	1 (3.2)	3 (9.7)	
Unknown	3 (9.7)	4 (6.4)	
**METASTASIS SITE**			
Lung	20 (64.5)	21 (67.7)	*P* = 0.138
Liver	7 (22.6)	12 (38.7)	*P* = 0.168
Kidney	14 (45.1)	9 (29.0)	*P* = 0.188
Bone	9 (29.0)	8 (25.8)	*P* = 0.775
Other	13 (41.9)	17 (54.8)^*^	*P* = 0.309

* *Four patients had brain metastasis*.

*ECOG, Eastern Cooperative Oncology Group; KPS, Karnofsky Performance Status; MSKCC, Memorial Sloan-Kettering Cancer Center; IMDC, International Metastatic Renal Cell Carcinoma Database Consortium. Data were presented as mean (standard deviation) for continuous variables or number (frequency) for categorical variables*.

### Clinical outcomes of the overall sequence

The median duration of pazopanib treatment was 8.1 months (Interquartile Range IQR 5.3–12.7); nine patients (31%) required a dose reduction, due to toxicity in 7 cases. The main cause of discontinuation was disease progression (87.2%), followed by toxicity (6.4%). At the time of the analysis, everolimus treatment was still ongoing in 4 patients, the median treatment duration was 4.4 months (IQR 3.4–6.5); five patients needed dose reduction for toxicity. Again, disease progression was the main cause for discontinuation (77.8%), followed by toxicity (18.5%).

**Figure 2:**
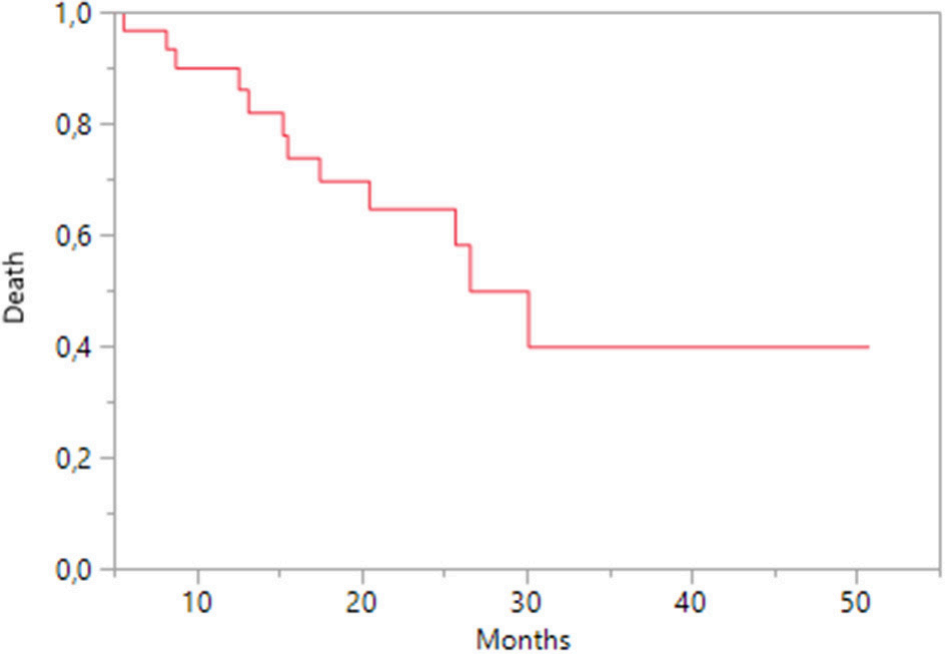
Kaplan-Meier estimates of Overall Survival for sequence.

OS for sequence was 26.5 months (95% CI 17.4-nc; Figure 2); OS was affected by the presence of metastases in the lung (HR 3.59; 95% CI 3.57-nc; *p* = 0.001) or liver (HR 6.4, 95% CI 1.17–34.98, *p* = 0.034) during everolimus treatment; age, previous nephrectomy, KPS status and histology did not correlate with it.

During pazopanib treatment, fatigue (32.2%), hypertension (48.4%), diarrhea (16.1%), thyroid disorders (19.3%), and hematological disorders (16.1%), including thrombocytopenia, leukopenia and anemia, were reported (Table 2). With everolimus, patients experienced diarrhea (14.3%), anemia (32.2%), and metabolic disorders, including hyper-triglyceridemia (16.1%), hypercholesterolemia (22.6%), and hyperglycemia (22.6%; Table 2).

**Table 2:** Adverse events.

**Adverse event**	**Pazopanib (*n* = 31)**	**Everolimus (*n* = 31)**
Fatigue	10 (32.2)	–
Hypertension	15 (48.4)	–
Diarrhea	5 (16.1)	4 (12.9)
Thyroid disorders	6 (19.3)	–
Mucositis	5 (16.1)	7
Rash	2 (6.5)	3
Hematological disorders	5 (16.1)	10 (32.2)
Thrombocytopenia	3	–
Leukopenia	1	–
Anemia	1	10
Metabolic disease	–	
Hyper-triglyceridemia		5 (16.1)
Hyper-cholesterolemia		7 (22.6)
Hyper-glycemia		7 (22.6)

### Clinical outcomes by treatment

**Figure 3:**
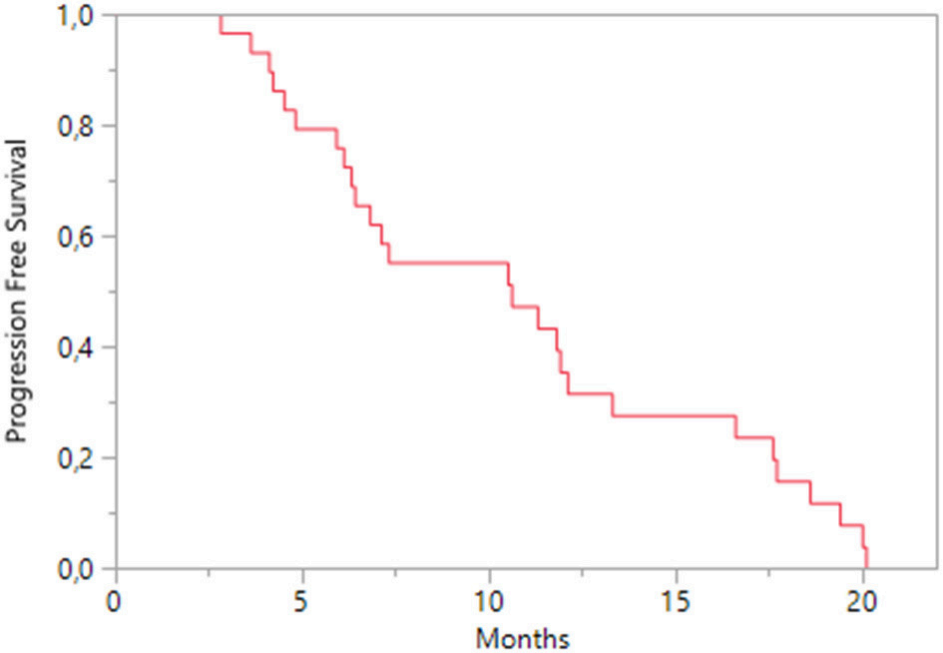
Kaplan-Meier estimates of Progression Free Survival on pazopanib treatment.

During pazopanib treatment, the best response was a partial response, achieved in 7 (24.1%) patients; a stable disease was obtained in 17 patients (58.6%) and 5 patients (17.3%) experienced a disease progression. The median PFS was 10.6 months (95% CI 6.3–12.1; Figure 3); accounting for performance status, patients with KPS ≥ 80 had a median PFS of 10.6 months (95% CI 6.4–13.3) and those with KPS < 80 a median of 4.2 months (95% CI 2.8–17.6). A Cox regression analysis indicated that PFS was positively influenced by lung metastasis (HR 0.293; 95% CI 0.105–0.757; *p* = 0.011) and clear cell histology (HR 0.32; 95% CI 0.12–0.94; *p* = 0.040); age, nephrectomy, health status (both KPS < 80 and ECOG ≥ 1), and other metastasis sites did not affect it.

**Figure 4:**
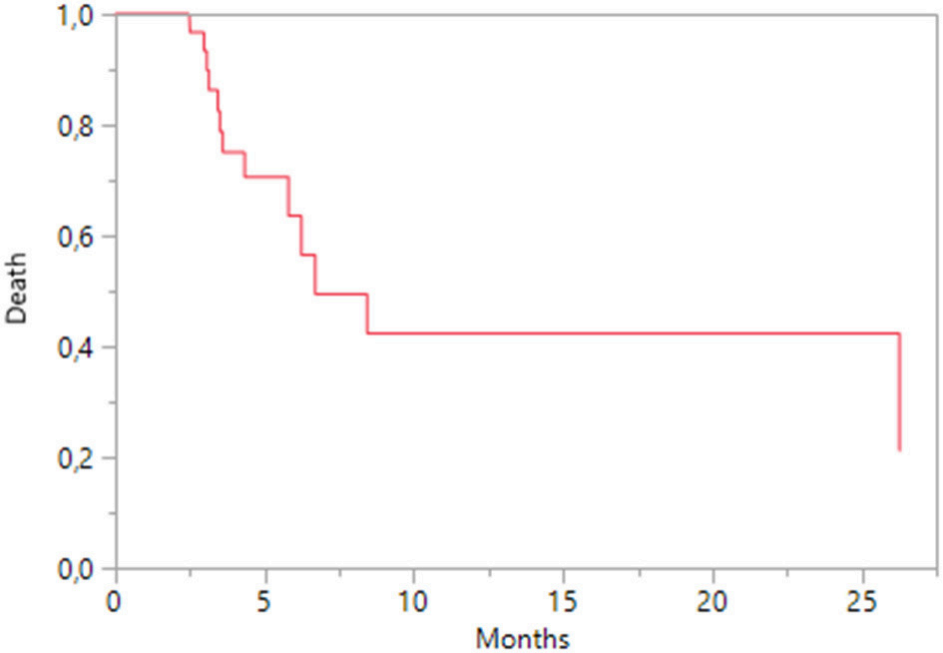
Kaplan-Meier estimates of Overall Survival on everolimus treatment.

**Figure 5:**
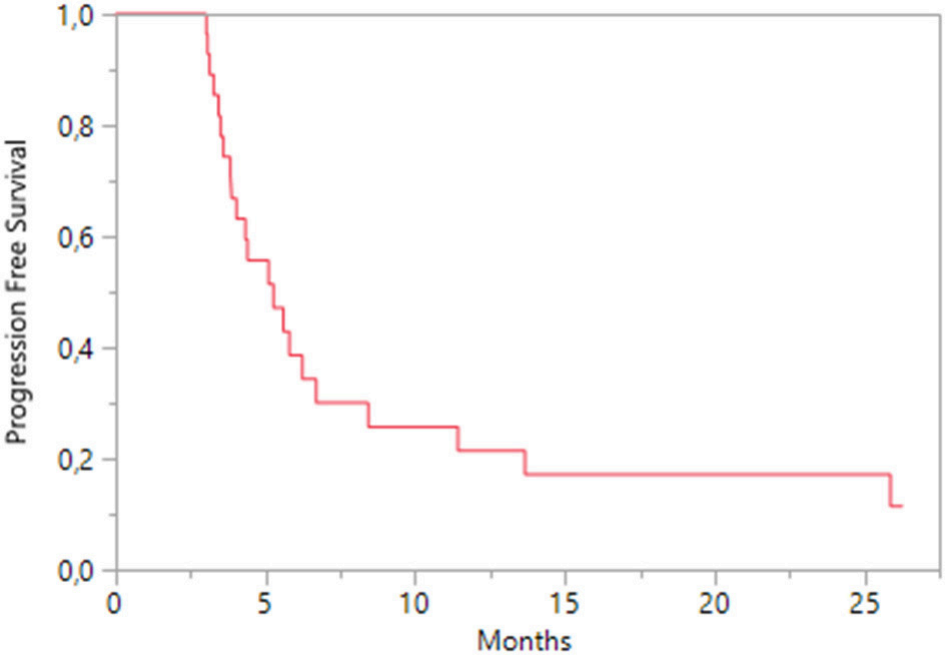
Kaplan-Meier estimates of Progression Free Survival on everolimus treatment.

During everolimus treatment, two patients achieved a partial response, while 13 patients (44.8%) had a stable disease and 14 patients (48.3%) a progression disease. Median OS was 6.7 months (95% CI 4.33-nc; Figure 4), while median PFS was 5.3 months (95% CI 3.8–6.7; Figure 5); according to KPS status, patients with KPS ≥ 80 had a median PFS of 4.6 months (95% CI 3.4–6.2) and those with KPS < 80 a median PFS of 5.3 months (95% CI 3.1–8.4). A Cox regression analysis showed that PFS was independent of age, previous nephrectomy, histology, KPS < 80, metastasis sites.

## Discussion

This observational study evaluated the clinical outcomes of a sequential treatment with pazopanib and everolimus in mRCC. As designed in real-world setting, the analysis included patients with poor performance status, comorbidities and metastases. Baseline characteristics of patients further worsened during the sequence, due to disease progression and deterioration of the global health status; for this reason, the direct comparison between pazopanib and everolimus treatment may be few informative. Other variables including age, risk status (both MDCC and IMDCC), and metastasis sites did not change.

The median duration of pazopanib treatment was similar to that reported in the phase III pivotal trial (7.4 months; Sternberg et al., 2010) and in the COMPARTZ trial (8.0 months; Motzer et al., 2013), and longer than in the real-world study based on US Oncology Network Database (5 months; Vogelzang et al., 2015). Although most of patients were older than 65 years and had multiple comorbidities, disease progression was the main cause of discontinuation, while toxicity more frequently determined a dose reduction. As previously reported (Cecere et al., 2016), dose reduction was not associated with a decreased efficacy, confirming that a personalized drug schedule and the persistence in therapy may enhance the therapeutic benefit. On average, everolimus treatment lasted 4.4 months and it was discontinued for disease progression in most cases, while toxicity was reported in five patients. Vogelzang et al. described a persistence in everolimus treatment of 93 days, discontinued for toxicity in 45% of cases (Vogelzang et al., 2015); in the RECORD-1 trial everolimus therapy lasted 141 days and interruption was related to adverse events in 35% of patients (Motzer et al., 2010).

The median OS (26.5 months) for sequence was longer than those reported in previous sequential studies, in the RECORD-1 study (14.4 months; Motzer et al., 2010) and in the analysis from the US Oncology Network Database (16 months; Vogelzang et al., 2015), and was similar to that we previously achieved in patients treated only with pazopanib (Cecere et al., 2016). Therefore, a supplemental therapy with everolimus may offer a clinical benefit for patients who needed to discontinue pazopanib, because of excessive toxicity. Among risk factors that could influence OS, the presence of metastases in lung and liver had a negative impact in the second line treatment with everolimus. Pal et al. recently accounted for negative prognostic factors the presence of higher tumor grade and lung, bone, or liver metastasis in patients with mRCC treated with angiogenesis inhibitors (Pal et al., 2017b).

The adverse event profile of two treatments was quite different: hypertension, hypothyroidism, and fatigue were observed only with pazopanib, whereas anemia and metabolic disorders were commonly reported with everolimus. Thus, patients showing intolerance to pazopanib may preferentially switch to a mTOR inhibitor and avoid potential for cumulative toxicity associated with sequential VEGFR-TKI treatment (D'Aniello et al., 2014a,b).

When each treatment was separately considered, most patients responded to both treatment, showing partial response or stable disease as the best response. Median PFS with pazopanib was 10.6 months; other real-world studies reported 13.7 months in the MD Anderson Cancer Center study (Matrana et al., 2013), 13.0 months in the Christie study (Galvis et al., 2013), 8.5 months in the US Oncology Network Database study (Vogelzang et al., 2015), and 12.7 months in Cecere et al (Cecere et al., 2016). The differences in the median PFS across studies may be explained by different operational definitions of PFS in terms of timing and frequency of disease assessment. Clear cell histology and metastasis localized in the lung represented favorable prognostic factors for PFS. Few cases on pazopanib efficacy in reducing lung metastases are described in literature. In a patient with inguinal epithelioid sarcoma, a clear reduction in the size of the pulmonary metastases was shown after 2.5 months of treatment with pazopanib and no development of new lesions was observed (Irimura et al., 2015). Another patient with metastatic pulmonary epithelioid hemangioendothelioma to the cervical and mediastinal lymph nodes, lungs and liver, treated with pazopanib for more than 2 years achieved a complete metabolic response in the mediastinum and lungs and long-lasting stable disease (Semenisty et al., 2015). As shown by our data, the OS of second-line treatment may be negatively affected by the persistence of lung metastases; therefore, further data on pazopanib activity on lung metastases in mRCC patients should be advisable. It has been already shown that the management of pulmonary metastases by surgical resection could provide long-lasting freedom from malignant disease and improve the 5-year survival (Pfannschmidt et al., 2012).

Everolimus treatment had a median PFS of 5.3 months, similar to those reported in the RECORD-1 study (5.5 months; Motzer et al., 2010) and in real-world (5.7 months; Vogelzang et al., 2015). The estimates of PFS were not significantly associated with clinical or disease characteristics during everolimus treatment. Hematological and metabolic toxicities were prevalently reported during this second treatment, and 12.9% of patients had diarrhea. This favorable tolerability was described in patients with a poor global health status, in presence of comorbidities and metastases. Everolimus is the standard comparison arm for all recent trials evaluated new drugs for pretreated mRCC patients. To date the results of major clinical trials involving nivolumab, cabozantinib, and lenvatinib plus everolimus, improved response rates (RR) and OS (Motzer et al., 2015; Mennito et al., 2016): these results dramatically change the therapeutic sequence in second-line setting. In the new treatment sequence, everolimus could be used in second-line therapy setting, in combination with levantinib for patients with a rapidly progressive high-volume disease considering the higher response rates (43%) and PFS of the combination, or for patients unfit for immunotherapy or antiangiogenic TKI. New clinical trials are needed to evaluate the efficacy of everolimus after disease progression following nivolumab and cabozantinib.

The main limitation of the study was the population size which limited the generalizability of results; however, our data were consistent and comparable with previous results reported in both randomized trials and real-world studies. Further analyses with a larger cohort will better elucidate the relationships between clinical and disease characteristics and outcomes of the sequential therapy.

## Conclusion

These data confirmed that pazopanib was effective, even in reduced dosing, and well tolerated and suggested that everolimus may represent an opportunity to continue a therapy when patients cannot further tolerate angiogenesis inhibitors or develop a resistance. Overall, the sequential therapy showed favorable clinical outcomes and a good safety profile, and may be feasible even for elderly patients or with multiple comorbidities. The choice of second-line treatment in the new therapeutic paradigm is dramatically changed with the approval of new drugs, such as nivolumab and cabozantinib. The next step in optimizing mRCC management would be the identification of new prognostic and predictive factors to detect a personalized sequence for each patient.

## Author contributions

SR, CD, GI, MP, MB, SP, GF, and CC substantially contributed to the conception of the work, data acquisition and interpretation. All the authors revised the work for important intellectual content and approved the final version to be published. They agree to be accountable for all aspects of the work in ensuring that questions related to the accuracy or integrity of any part of the work are appropriately investigated and resolved.

### Conflict of interest statement

The authors declare that the research was conducted in the absence of any commercial or financial relationships that could be construed as a potential conflict of interest.
